# Experimental Study on the Impact Resistance of UHMWPE Flexible Film Against Hypervelocity Particles

**DOI:** 10.3390/polym18020161

**Published:** 2026-01-07

**Authors:** Chen Liu, Zhirui Rao, Hao Liu, Changlin Zhao, Yifan Wang, Aleksey Khaziev

**Affiliations:** 1 School of Physics, Harbin Institute of Technology, Harbin 150001, China; liuchen2016@hit.edu.cn (C.L.); m18345543049@163.com (Z.R.); 2 School of Mechanical Engineering, Xi’an Jiaotong University, Xi’an 710049, China; 3 Department of Civil Engineering, Faculty of Engineering, Universiti Putra Malaysia, Serdang 43400, Selangor, Malaysia; gs69966@student.upm.edu.my; 4 Department of Mechanical Engineering Technologies, Bauman Moscow State Technical University, Moscow 105005, Russia; wangyf_wangyf@163.com; 5 Center for Materials Technologies, Skolkovo Institute of Science and Technology, Moscow 121205, Russia

**Keywords:** UHMWPE, flexible film, hypervelocity impact, impact resistance, damage, microparticle, spacecraft shielding

## Abstract

The increasing threat posed by micrometeoroids and orbital debris to in-orbit spacecraft necessitates the development of lightweight and deformable shielding systems capable of withstanding hypervelocity impacts. Ultra-high-molecular-weight polyethylene (UHMWPE) films, owing to their high specific strength and energy-absorption capacity, present a promising candidate for such applications. However, the hypervelocity impact response of thin, highly oriented UHMWPE films—distinct from bulk plates or composites—remains poorly understood, particularly for micron-scale particles at velocities relevant to space debris (≥8 km/s). In this study, we systematically investigate the impact resistance of 0.1 mm UHMWPE films using a plasma-driven microparticle accelerator and a hypervelocity dust gun to simulate impacts by micron-sized Al_2_O_3_ and Fe particles at velocities up to ~8.5 km/s. Through detailed analysis of crater morphology via scanning electron microscopy, we identify three distinct damage modes: plastic-dominated craters (Type I), fracture-melting craters (Type II), and perforations (Type III). These modes are correlated with impact energy and particle size, revealing the material’s transition from large-scale plastic deformation to localized thermal softening and eventual penetration. Crucially, we provide quantitative penetration thresholds (e.g., 2.25 μm Al_2_O_3_ at 8.5 km/s) and establish a microstructure-informed damage classification that advances the fundamental understanding of UHMWPE film behavior under extreme strain rates. Our findings not only elucidate the energy-dissipation mechanisms in oriented polymer films but also offer practical guidelines for the design of next-generation, flexible spacecraft shielding systems.

## 1. Introduction

With the continuous increase in launch frequency and on-orbit duration, spacecraft face growing risks of impacts from micrometeoroids and orbital debris (MMOD). In particular, alumina (Al_2_O_3_) particles generated from solid propellant residues or fragmentation events pose a severe threat due to their high hardness and density [1,2,3,4]. When traveling at velocities of several to tens of kilometers per second, these particles can cause localized perforation, erosion, and the generation of secondary debris clouds upon striking spacecraft surfaces, potentially damaging critical subsystems [3,4]. Traditional shielding approaches, such as Whipple shields and hybrid multilayer structures, can mitigate these threats to some extent but often entail significant mass penalties and spatial constraints [1,2,3]. Previous studies [1,2,3,4,5,6,7,8] and engineering designs have primarily relied on projectile impact tests or Whipple configurations to simulate such collisions. However, these tests typically use millimeter-scale projectiles at medium-to-high velocities, with target materials mainly in the form of plates or woven composites. For example, Rogers et al. [7,8] employed ~10 mm aluminum spheres to impact UHMWPE (ultra-high-molecular-weight polyethylene) and HDPE (high-density polyethylene) plates at 2–6.5 km/s, analyzing parameters such as fragment cloud velocity, mass loss, and perforation size. While such studies provide valuable insights for spacecraft shielding design, experimental data remain extremely limited for micron-sized particle impacts on thin films, particularly at velocities exceeding the upper limits of most test facilities. Critically, the impact mechanics of micron-scale particles on thin, free-standing films differ fundamentally from those of millimeter-scale projectiles on thick plates or Whipple shields. In Whipple configurations, the primary mechanisms include projectile fragmentation, shock propagation, and secondary debris cloud formation, which are strongly influenced by the spacing and thickness of the bumper and rear walls. In contrast, micron-scale impacts on sub-millimeter films are governed by localized strain-rate effects, adiabatic heating, and microstructural deformation mechanisms such as chain drawing and fibrillation, which cannot be directly extrapolated from macroscopic tests. Moreover, at velocities approaching ~8.5 km/s—surpassing the typical range of light-gas guns—thermal softening and melting become significant, phenomena that are often negligible in lower-velocity, larger-scale impacts. Therefore, studying micron-scale Al_2_O_3_ impacts on thin UHMWPE films at such velocities is essential to uncover material-specific, strain-rate-dependent failure modes that are otherwise masked in conventional Whipple tests. Furthermore, the impact mechanisms of micron-scale debris differ significantly from those of millimeter- or centimeter-scale projectiles, and the interaction mechanisms within flexible thin-film materials remain poorly understood [9,10]. Consequently, existing models of polymer impact behavior—largely derived from millimeter-scale projectile tests on plates or composites—do not adequately capture the damage evolution of oriented UHMWPE films under hypervelocity (≥8 km/s) and micron-scale impact conditions. Specifically, three critical gaps remain: (i) Lack of penetration thresholds for micron-scale particles on sub-millimeter films, (ii) Unclear transition mechanisms between plastic deformation, thermal softening, and perforation in highly oriented polymer films, and (iii) Absence of a microstructure-informed damage classification that can guide the design of flexible, multi-layer shielding systems.

In terms of material selection, UHMWPE has attracted widespread attention due to its excellent specific strength, elongation at break, and energy-absorption capacity [11,12,13]. Recent advances in the fabrication of highly oriented, high-draw-ratio, and low-entanglement UHMWPE films have endowed the material with unique chain alignment and crystalline structures [11,12], leading to thermal and mechanical behaviors distinct from bulk plates or woven laminates [13,14,15]. Studies have shown that such films can dissipate energy effectively under high-strain-rate loading through chain stretching, fibrillation, and molecular-scale rearrangements [11,12,14]. For instance, Kim et al. [11] fabricated disentangled UHMWPE films with a draw ratio of ~200, reporting a thermal conductivity ≥ 50 W·m^−1^·K^−1^, thereby demonstrating the influence of enlarged crystalline domains and high chain orientation on phonon mean free paths. Pan et al. [15] prepared ultra-stretched UHMWPE/graphene composite films to enhance thermal conductivity and optical transparency, revealing tunable film properties under oriented stretching even outside impact contexts. Regarding strain-rate sensitivity, studies by scholars [16] have investigated UHMWPE under combined tensile and dynamic loading, examining stress–strain responses and their implications for high-velocity impact performance. Nevertheless, experimental reports on UHMWPE films under hypervelocity conditions remain scarce [7,9,14]. Although UHMWPE films, owing to their unique microstructure and mechanical properties, offer potential advantages for lightweight spacecraft shielding, their damage evolution and protective mechanisms under hypervelocity microparticle impacts require further systematic investigation.

Most existing hypervelocity impact experiments rely on light gas guns with millimeter-scale projectiles at velocities of 2–6.5 km/s, rarely exceeding 7 km/s [7,9,14]. Research on micron-scale particles has instead depended on electrostatic accelerators, plasma-driven accelerators, and laser/optical-based devices [17,18,19,20,21]. For example, the SSERVI-IMPACT electrostatic dust accelerator at the University of Colorado can accelerate micron- and submicron-sized particles to 10–30 km/s [18,19], while laser- and optically driven accelerators have also been increasingly reported in recent years [13,20,21]. These facilities not only enable controlled single-particle launches but also support diagnostic methods such as impact plasma emission, secondary electron detection, and surface morphology analysis [22,23,24,25]. However, detailed characterizations of penetration thresholds, deformation mechanisms, fragment cloud evolution, and microscopic surface damage features (e.g., microcracks, melting, or carbonization) under extreme velocities remain incomplete. In particular, experimental data on micron-scale Al_2_O_3_ impacts on ultrathin UHMWPE films are still scarce, especially in the velocity range of 8–10 km/s or higher.

In contrast to prior investigations on UHMWPE plates or composites, which primarily involve millimeter-scale projectiles at velocities up to ~6.5 km/s [7,8], the present study focuses on highly oriented, low-entanglement UHMWPE films with a thickness of only 0.1 mm, subjected to micron-scale particle impacts at velocities extending up to ~8.5 km/s. Such films exhibit markedly different chain alignment and crystallographic characteristics compared to their bulk counterparts, leading to distinct deformation and energy-dissipation mechanisms under extreme strain rates [11,12,14]. While existing literature provides valuable data on fragment clouds and perforation thresholds for plate-like UHMWPE targets, systematic studies on the damage morphology, penetration thresholds, and microstructural response of ultrathin UHMWPE films under true hypervelocity conditions remain notably absent. The specific contributions are: (1) Determine quantitative penetration thresholds as a function of particle size and velocity, (2) Elucidate the underlying deformation mechanisms—including strain-rate-dependent fracture and adiabatic thermal softening—through detailed crater morphology analysis, and (3) Propose a three-type damage classification that links impact conditions to failure modes. By doing so, this work not only extends the velocity range and particle-size resolution beyond prior studies (e.g., [7,9]) but also provides mechanism-based guidelines for the design of lightweight, deformable spacecraft shielding systems that leverage the unique energy-absorption capabilities of oriented UHMWPE films.

## 2. Materials and Methods

### 2.1. UHMWPE Film

**Figure 1:**
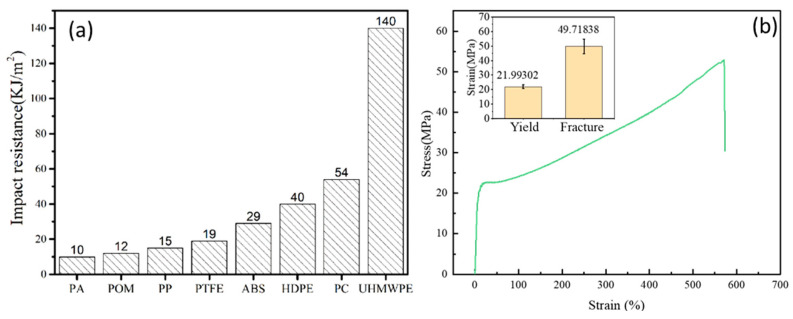
(**a**) Comparison of impact properties of several polymer materials [27]. Here, PA refers to polyamide, POM to polyoxymethylene, PP to polypropylene, and PTFE to polytetrafluoroethylene. (**b**) Tensile properties of UHMWPE film.

UHMWPE generally refers to linear polyethylene with a viscosity-average molecular weight above 1.5 million and no side chains. The extremely high-molecular-weight and long-chain segments of UHMWPE tend to cause severe entanglement at the molecular scale, resulting in dense physical entanglements. These characteristics enhance intermolecular interactions, while the ultra-long chains allow efficient load transfer, thereby endowing UHMWPE with outstanding impact resistance [12]. Among engineering plastics, UHMWPE exhibits exceptional toughness and impact strength. As shown in Figure 1a, which compares the impact performance of several engineering plastics, the impact strength of UHMWPE far exceeds that of polycarbonate (PC), a material known for its toughness, and is approximately five times that of the commonly used acrylonitrile butadiene styrene (ABS) plastic [26].

The UHMWPE film used in this study was a commercially available, gel-spun and ultra-drawn film (GUR^®^ UHMWPE 4113). The film has a viscosity-average molecular weight of approximately 3.7 × 10^6^ g/mol and a density of 0.93 g/cm^3^. Based on the supplier’s specifications and typical processing routes for such films, the draw ratio is estimated to be >150, resulting in a high degree of molecular orientation and crystallinity (typical crystallinity for similarly processed films ranges between 75–85%). These structural characteristics—high chain alignment, reduced entanglement, and enlarged crystalline domains—are known to significantly enhance the film’s energy-absorption capacity and strain-rate sensitivity under impact loading [11,12]. A 0.1 mm-thick UHMWPE film was selected for this study to facilitate the subsequent design of deformable protective structures. The film appears semi-transparent, with a smooth microstructural surface, making it suitable for subsequent damage characterization.

To obtain the mechanical properties of the UHMWPE film, uniaxial tensile tests were conducted. The specimen dimensions were 150 mm in length, 10 mm in width, and 0.1 mm in thickness, with a gauge length of 50 mm. The loading speed was set at 600 mm/min. Figure 1b shows the mechanical properties of UHMWPE film, which exhibited an elastic strength limit of approximately 22.0 MPa and a plastic fracture strength of about 49.7 MPa, demonstrating a clear elastic–plastic behavior with pronounced strain hardening. The measured fracture strength is lower than the values typically reported for highly drawn UHMWPE fibers or specialized laboratory-grade films. This difference can be attributed to several factors: (i) the tensile test was performed on a free-standing thin film (0.1 mm) without lateral constraint, which may promote earlier necking and failure compared to constrained fiber bundles or thicker laminates, and (ii) although the film is gel-spun and drawn, commercial films may exhibit slightly lower orientation or contain processing-induced micro-defects (e.g., minor surface irregularities or localized entanglement) that reduce the macroscopic tensile strength while still retaining high impact energy-absorption capacity.

### 2.2. Al_2_O_3_ Particles and Fe Powder

In this study, micron-sized Al_2_O_3_ particles were selected to simulate micrometeoroids and orbital debris in space. The particle size distribution of the Al_2_O_3_ samples was analyzed using a laser particle size analyzer (Bettersize 2600, Bettersize Instruments, Dandong, China). The particle size distribution is calculated from the scattering spectrum generated by parallel light irradiating the particles. A small amount of Al_2_O_3_ powder was dispersed in ethanol, ultrasonicated for 10 min, and then tested with the Bettersize 2600 under circulation and ultrasonic conditions. The sample particle size distribution was subsequently obtained.

**Figure 2:**
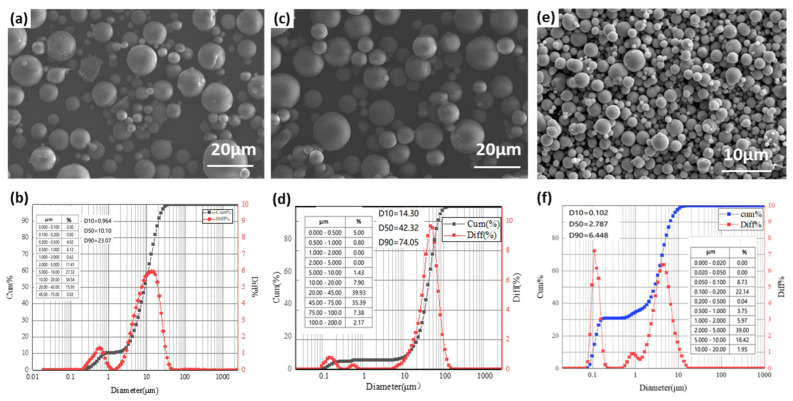
Morphology and particle size distribution curves of (**a**,**b**) Al_2_O_3_ with 10 μm, (**c**,**d**) Al_2_O_3_ with 40 μm, (**e**,**f**) Fe with 10 μm.

Figure 2 presents scanning electron microscopy (SEM) images and particle size distributions of two different Al_2_O_3_ particle groups. As shown in Figure 2a,c, the Al_2_O_3_ particles exhibit good sphericity and smooth surfaces, though their sizes are not entirely uniform, with smaller particles dispersed among larger ones. The particle size distribution curves in Figure 2b,d show approximately normal distributions, with median diameters (D50) of 10.1 μm and 42.3 μm, respectively.

Due to the limitations of the acceleration devices, ferrum (Fe) particles were employed when using the hypervelocity dust gun to accelerate fine particles. Figure 2e shows the SEM morphology of Fe particles, which display good sphericity and smooth surfaces. The overall particle sizes vary, with maximum and minimum diameters of approximately 4.5 μm and 0.4 μm, respectively. As shown in Figure 2f, the particle size distribution of Fe powder exhibits two main peaks, centered at ~0.1 μm and ~4.5 μm, both with narrow distributions, indicating that the particle sizes are concentrated in these two ranges. In addition, a small fraction of particles with diameters around 0.9 μm is also present.

### 2.3. Hypervelocity Impact Experiments

#### 2.3.1. Plasma-Driven Microparticle Accelerator

**Figure 3:**
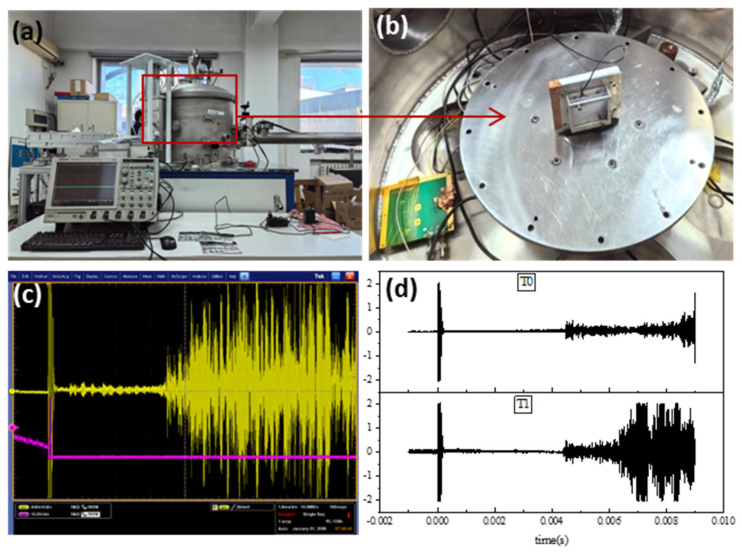
(**a**) Plasma-driven micro-debris accelerator, (**b**) Sample installation, (**c**) signal collected by plasma-driven micro-debris accelerator and (**d**) signal collected by T0 and T1 experiments and time diagram.

Plasma-driven microparticle accelerator was shown in Figure 3a, at the Technology and Engineering Center for Space Utilization, Chinese Academy of Sciences. The accelerator mainly consists of a capacitor bank, switches, a pulsed gas valve, inner and outer electrodes, a compression coil, a control circuit, and a vacuum system. The vacuum level was below 5 × 10^−3^ Pa. For ground-based simulations of space debris impacts, the plasma-driven accelerator is capable of accelerating microparticles ranging from a few micrometers to several hundred micrometers in size to velocities of 1–15 km/s. Depending on the experimental requirements, both metallic and nonmetallic particles can be selected. Moreover, the device allows group emission of microparticles with varied shapes, sizes, materials, and velocities, making it particularly suitable for studying the impact effects of micrometer-scale space debris [4].

Al_2_O_3_ particles with diameters of 10 μm and 40 μm were selected as impact projectiles. The targets were UHMWPE films with a thickness of 0.1 mm, cut into square samples of 5 cm × 5 cm, and fixed onto the sample holder, as shown in Figure 3b.

The particle velocity was primarily measured using piezoelectric sensors. Figure 3c shows the signal collected during one experiment: the initial signal was triggered at the moment of circuit closure, while the terminal signal was generated when the particles struck the piezoelectric sensor mounted behind the target plate. Given the flight path length of 5.4 m, the impact velocity of the particles was calculated from the time interval between the two signals. As shown in Figure 3c, the shortest time interval was approximately 0.0064 s, corresponding to a maximum particle velocity of ~8.5 km/s. After about 0.0034 s, additional peaks appeared, indicating that while a small fraction of particles reached velocities up to 8.5 km/s, the majority traveled at velocities between 600 m/s and 1600 m/s.

It should be noted that the terminal signal requires particles to directly strike the sensor. When the UHMWPE sample was installed, the collected signals were either difficult to obtain or contained significant errors. To ensure more accurate velocity measurements, tests were first conducted without UHMWPE films to record reliable signals. The impact velocity under corresponding conditions was then determined using these measurements, and the UHMWPE targets were subsequently installed for impact testing. Figure 3d presents the signals from two repeated experiments under identical conditions. The comparison of experiments T0 and T1 demonstrates that the maximum velocities were consistent, confirming the repeatability of the acceleration device. Therefore, the velocities derived from tests without the UHMWPE targets were used as the reference impact velocities.

Three experimental runs were conducted using the plasma-driven micro-debris accelerator, with the measured velocities summarized in Table 1. Experiments performed without targets were labeled A0, B0, and C0 to determine the particle velocity distributions under the corresponding conditions. The experiments with targets were designated A, B, and C, which shared the same accelerator settings as A0, B0, and C0, respectively. Thus, the particle velocities in A, B, and C correspond directly to those in A0, B0, and C0. Specifically, 40 μm Al_2_O_3_ particles were used in experiments A0, B0, A, and B, while 10 μm Al_2_O_3_ particles were employed in experiments C0 and C.

Given the nature of powder-based acceleration, precise selection of individual particle sizes was not feasible; instead, a relatively narrow size distribution was targeted for each experimental run (see Section 2.2). Consequently, each data point corresponds to a separate experimental run under nominally identical accelerator settings (capacitor bank charge, gas pressure, etc.). The impacts were single-particle events within a given run, though multiple particles were accelerated per shot, resulting in a sparse distribution of impact sites on the sample surface (typically several craters per cm^2^). All impacts were conducted at a normal incidence angle (90°) relative to the film surface, ensured by aligning the sample holder perpendicular to the particle beam axis. The vacuum levels during operation were maintained below 5 × 10^−3^ Pa for the plasma-driven accelerator and below 5 × 10^−4^ Pa for the high-velocity dust chamber, minimizing aerodynamic drag and particle-gas interactions. Despite the challenge of achieving perfectly identical conditions across runs due to the stochastic nature of powder acceleration, the consistency of velocity distributions between target-free calibration runs (A0, B0, C0) and corresponding impact runs (A, B, C) confirms the repeatability of the acceleration process under fixed equipment parameters.

#### 2.3.2. High-Velocity Dust Chamber

**Figure 4:**
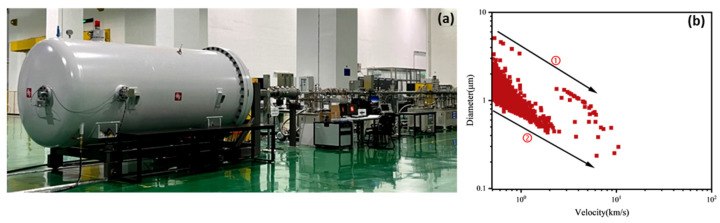
(**a**) High-speed dust chamber structure and (**b**) Fe particle velocity-size relationship in the F experiment.

A high-velocity dust chamber developed by Harbin Institute of Technology was employed to accelerate Fe particles, as shown in Figure 4a. The high-velocity dust chamber mainly consists of a high-velocity dust accelerator, a high-velocity dust impact chamber, a dust selection system, a sample stage, an in situ analysis system, as well as beamline and vacuum maintenance subsystems. The dust accelerator is the core component of the system, comprising a high-voltage generator, dust source, vacuum pipeline, and SF_6_ recovery system. By applying electrostatic acceleration, the accelerator imparts high velocity to charged dust particles originating from the dust source, thereby generating a stable high-speed dust flow. The vacuum level was below 5 × 10^−4^ Pa.

Fe particles were selected for hypervelocity impact experiments on UHMWPE films with a thickness of 0.1 mm. A single experiment was conducted using the high-velocity dust chamber and designated as Experiment F. Figure 4 shows the relationship between particle size and velocity for Fe particles in this experiment.

As shown in Figure 4, larger Fe particles generally exhibited lower velocities. Based on the particle size–velocity distribution, two boundary lines (lines 1 and 2) were drawn, which encompass the velocity range for the Fe particles. For particles of the same size, those closer to line 1 had higher velocities, whereas most particles were closer to line 2, indicating that the majority of Fe particles were accelerated to relatively low velocities ranging from approximately 0.2 to 2 km/s, with a few reaching higher velocities between 2 and 10 km/s. For example, Fe particles with diameters between 0.95 and 1.05 μm exhibited velocities ranging from 0.18 to 7.4 km/s. Most particles were located near line 2, corresponding to velocities of 0.18–0.76 km/s, while a small fraction near line 1 reached velocities up to 7.4 km/s.

The time-of-flight velocimetry system is an integrated component of the accelerator and is factory-calibrated using standard reference particles. Although the detailed calibration protocol is proprietary, the system’s timing resolution is ≤0.1 μs, and the flight-path length is known to within ±1 cm. Propagating these uncertainties yields an estimated velocity error of approximately ±3% for velocities above 1 km/s, corresponding to roughly ±0.3 km/s at 8.5 km/s. This uncertainty level is consistent with reported accuracies of similar plasma-driven accelerators, e.g., Refs. [17,19]. It should be noted that the velocity distribution within each run (see Figure 3c) reflects the intrinsic dispersion of the acceleration process, not measurement error. The reported velocity ranges (e.g., 0.6–8.5 km/s) encompass this natural spread, and the corresponding kinetic-energy calculations are therefore presented as range-based estimates rather than precise single-particle values.

## 3. Results

**Figure 5:**
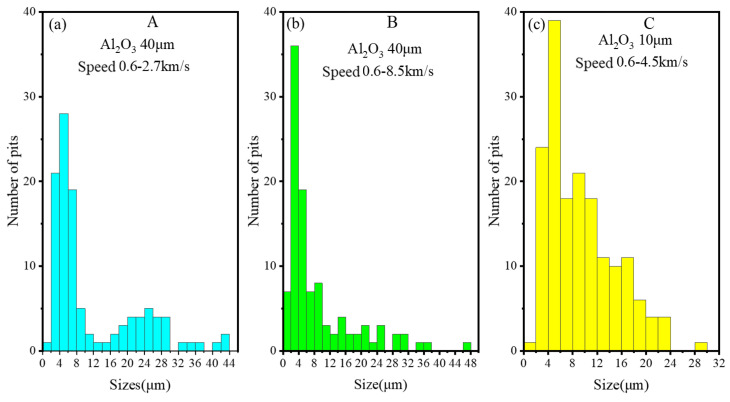
Crater/hole size-number diagram for the three experimental groups: (**a**) Experiment A, (**b**) Experiment B, (**c**) Experiment C.

The sizes of craters/holes on UHMWPE films after hypervelocity impact tests were statistically analyzed. The microcrater size distribution is shown in Figure 5. In Experiment A, Al_2_O_3_ particles had velocities of 0.6–2.7 km/s, and the diameters of the resulting craters/holes ranged from 0 to 44 μm, with a higher frequency observed for craters approximately 4–6 μm and 20–30 μm in diameter. In Experiment B, Al_2_O_3_ particle velocities ranged from 0.6–8.5 km/s, with crater diameters also ranging from 0–44 μm. The overall trend indicated that larger craters were less frequent. The trend in Experiment C was similar to that of Experiment B, but the crater size range was smaller (0–30 μm) due to the smaller particle diameter.

**Figure 6:**
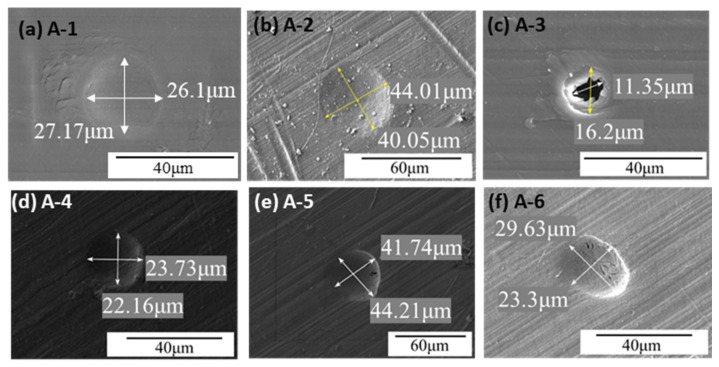
Typical morphology of the impact crater/hole in Experiment A.

In Experiment A (0.6–2.7 km/s) with 40 μm Al_2_O_3_ particles, the SEM images of the UHMWPE film surface after impact are shown in Figure 6. Crater A-1 is circular with a diameter of ~26 μm; layered folds are observed on one side of the crater, indicating energy absorption via extensive plastic deformation, and no fragmented particles remain inside. Crater A-2 is roughly circular with a diameter of ~44 μm, containing numerous fragmented particles both inside and around the crater. Crater A-3 is a perforation; the surrounding region shows plastic deformation-induced bulges, with an external diameter of ~16.2 μm and an irregular internal perforation of ~11.35 μm. Few fragmented particles are observed inside. Crater A-4 is irregularly circular, with maximum and minimum diameters of 44.21 μm and 41.74 μm, respectively, containing multiple microcracks. Crater A-5 is circular with a diameter of ~22 μm, appearing deeper than A-1 and A-3. Crater A-6 is elliptical, with long and short axes of 29.63 μm and 23.3 μm, respectively, and contains multiple microcracks similar to A-4.

**Figure 7:**
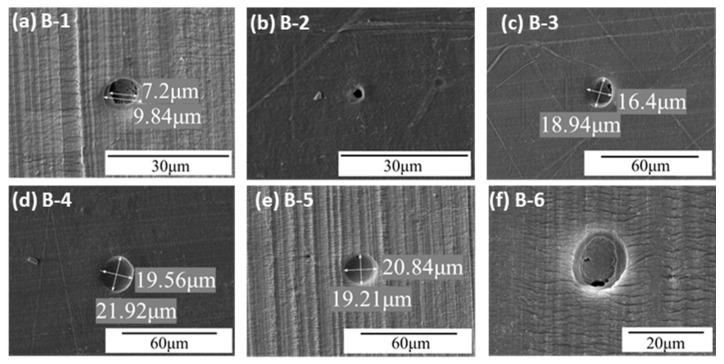
Typical morphology of the impact crater/hole in Experiment B.

In Experiment B (0.6–8.5 km/s) with 40 μm Al_2_O_3_ particles, the post-impact SEM images are shown in Figure 7. Crater B-1 contains two distinct internal features; plastic fracture is evident inside, with an external diameter of ~9.84 μm and an internal diameter of 7.2 μm. Crater B-2 is a perforation with an external damaged region of ~4.32 μm and an internal perforation of ~2.25 μm. Crater B-3 is elliptical with multiple small internal pits. Crater B-4 is irregularly circular, with maximum and minimum diameters of 21.92 μm and 19.53 μm, respectively. Crater B-5 is circular and similar in size to B-74, but with a deeper internal pit. Crater B-6 is elliptical, with external axes of 16.57 μm and 14.68 μm and internal axes of 13.02 μm and 9.37 μm, showing clear features of compression fracture and melting.

**Figure 8:**
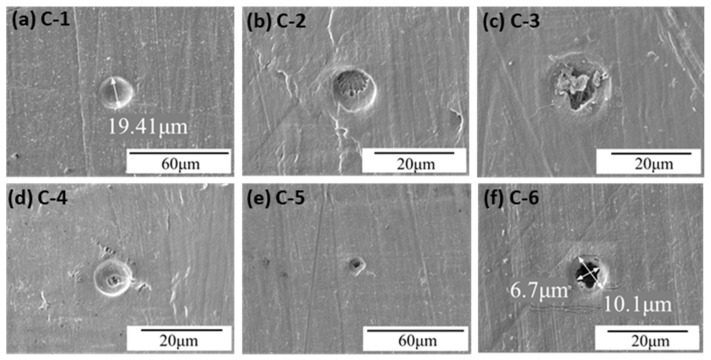
Typical morphology of the impact crater/hole in Experiment C.

In Experiment C (0.6–4.5 km/s) with 10.1 μm Al_2_O_3_ particles, the SEM images of the UHMWPE film surface are shown in Figure 8. Crater C-1 is circular, with a diameter of ~19.41 μm. Crater C-2 has an outer circular pit of ~11.27 μm and an inner pit of ~7.4 μm exhibiting fracture features. Crater C-3 is circular, with large fragmented Al_2_O_3_ particles remaining inside; plastic deformation and compressive fracture are observed beneath the fragments, with outer and inner diameters of 14.46 μm and 9.3 μm, respectively. Crater C-4 is circular with a smaller internal pit, exhibiting rib-like tearing, with diameters of 9.37 μm and 2.74 μm for the outer and inner pits. Crater C-5 is a perforation with an irregular outer pit (~11.5 μm) and an internal diameter of ~4.76 μm. Crater C-6 is another perforation with outer and inner diameters of 10.1 μm and 6.7 μm, respectively.

**Figure 9:**
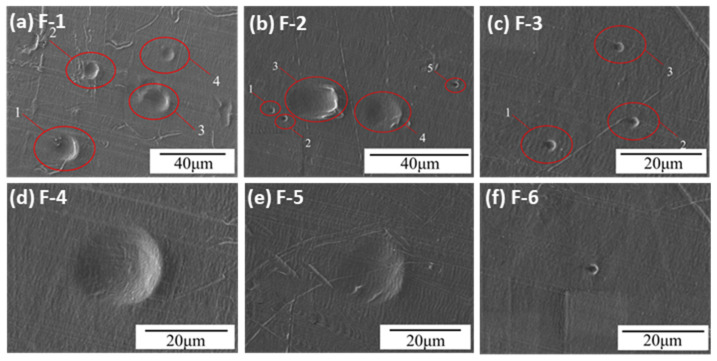
Typical morphology of the impact crater/hole in Experiment F.

For Experiment F, Fe particles were accelerated using the high-velocity dust chamber. Laser particle size analysis indicates that Fe particles are primarily around 0.1 μm and 4 μm. SEM images of the UHMWPE film after hypervelocity impact are shown in Figure 9. In Figure 9a–c, the impact craters are dense. In Figure 9a, four craters are observed, with diameters of 14.46, 10.06, 13.67, and 7.82 μm, all larger than the main particle size of 4 μm. In Figure 9b, five craters have diameters of 1.81, 1.86, 10.58, 11.7, and 1.98 μm; craters 3 and 4 exceed the main particle size, while craters 1, 2, and 5 are smaller. In Figure 9c, three craters range from 2.14 to 2.5 μm, representing smaller pits. Figure 9d–f show crater diameters of 19.66, 17.14, and 2.2 μm. Across these images, Fe particle impact craters are regularly circular; in Figure 9a, craters 1–3 exhibit clear plastic deformation, and no fragmented Fe particles remain on the surface. No perforations were observed in UHMWPE films impacted by Fe particles.

The observed crater morphologies can be understood in terms of the intrinsic microstructure and high-strain-rate behavior of highly oriented UHMWPE films. Unlike isotropic bulk polymers, the aligned molecular chains and reduced entanglement in these films facilitate energy dissipation through large-scale plastic drawing, fibrillation, and chain scission rather than brittle fracture [11,12]. The present study advances prior work in several key aspects:

(i) Material form: While Rogers et al. [7,8] examined monolithic UHMWPE plates impacted by ~10 mm spheres, our use of 0.1 mm free-standing films represents a distinct structural configuration relevant to deployable or conformal shielding.

(ii) Particle scale: The employment of micron-sized Al_2_O_3_ and Fe particles closely mimics the actual size distribution of micrometeoroid and orbital debris, providing data that are more directly applicable to real-space threats than millimeter-scale tests.

(iii) Damage classification: The proposed three-type crater classification offers a predictive framework for assessing the failure modes of flexible UHMWPE films under varying impact conditions, a step beyond purely descriptive reporting.

Accordingly, critical penetration diameters were determined statistically based on repeated impact tests (*n* = 5 for each particle-size group). For each velocity condition, the smallest particle diameter that produced through-thickness perforation was identified. Additionally, a logistic regression of penetration probability versus particle diameter was performed, and the diameter corresponding to 50% penetration probability is reported as the ‘critical penetration diameter’. For a 0.1 mm UHMWPE film, the critical penetration diameters for Al_2_O_3_ particles at velocities of 2.7 km/s, 4.5 km/s, and 8.5 km/s were 11.35 μm, 4.76 μm, and 2.25 μm, respectively.

## 4. Discussion

**Figure 10:**
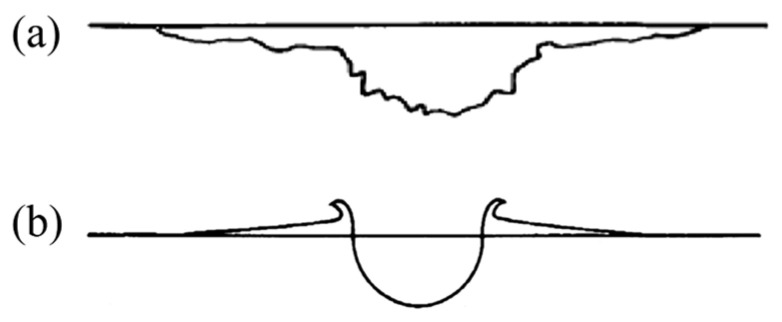
Schematic diagram of the impact crater formed in the material by hypervelocity impact [28]: (**a**) brittleness, (**b**) plasticity.

Figure 10 illustrates the characteristic impact craters formed on ductile and brittle materials under hypervelocity impact. Brittle materials exhibit large spallation zones due to the presence of numerous microcracks and defects on their surfaces. During hypervelocity impact, local stress can greatly exceed the actual elastic modulus, resulting in rapid localized failure and the formation of large spallation regions. In contrast, craters on ductile materials are generally semi-circular with protrusions caused by plastic deformation [28,29]. The craters/holes observed in this study on UHMWPE films differ distinctly from those of typical brittle and ductile materials. Multiple craters/holes from the three impact experiments were statistically analyzed and classified into three types.

(1) Type I Craters

These craters include A-1, A-2, A-4, A-5, and A-6 in Figure 6; B-3, B-4, and B-5 in Figure 7; and C-1 and C-2 in Figure 8. Type I craters are circular, with moderate plastic deformation around the crater. Some craters contain fragmented Al_2_O_3_ particles. They are typical plastic deformation craters formed by hypervelocity Al_2_O_3_ particles impacting UHMWPE films, inducing plastic deformation, melting, vaporization, and fragmentation of the Al_2_O_3_ particles.

(2) Type II Craters

Type II craters exhibit two distinct regions, as observed in B-1 and B-6 in Figure 7 and C-2 and C-4 in Figure 8. The outer region resembles Type I craters, formed by plastic deformation. The inner region exhibits fracture and even localized melting. The formation process can be inferred as follows: Al_2_O_3_ particles first contact the UHMWPE film and form a Type I crater through impact and wave interactions. Particles retaining residual velocity then deform the inner region beyond the material’s fracture strength, leading to fracture and, in some regions, thermal melting. Xie et al. [27] reported that UHMWPE under hypervelocity impact exhibits large-scale plastic deformation, thermal softening, and melting, effectively absorbing impact energy and enhancing protective performance.

(3) Type III Craters

Type III craters are an evolution of Type II craters. They also have two regions, but the inner region cannot fully absorb the particle’s kinetic energy, resulting in perforation. Examples include A-3 in Figure 6, B-2 in Figure 7, and C-5 and C-6 in Figure 8.

Notably, beyond merely cataloging crater dimensions, our analysis yields two key physical insights into the behavior of UHMWPE films under hypervelocity impact:

(i) Strain-rate-dependent failure transition: At lower velocities (Type I), the film deforms plastically via chain uncoiling and drawing, consistent with quasi-static tensile behavior. As velocity increases (Type II), the strain rate surpasses the polymer’s relaxation timescale, leading to brittle-like micro-fracture and fibrillation within the crater interior—a direct manifestation of strain-rate-induced embrittlement.

(ii) Localized thermal softening and melting: The clear evidence of melting in Type II craters indicates that, under micron-scale confinement, the impact energy is deposited within a volume too small for rapid heat dissipation, resulting in adiabatic temperature rises sufficient to melt UHMWPE (130–145 °C). This thermal softening reduces the local yield strength, facilitating further plastic flow and energy absorption—a mechanism rarely observed in thicker targets where heat is conducted away more efficiently. These insights underscore that the protective performance of thin UHMWPE films is governed not only by their high toughness but also by their ability to undergo strain-rate- and thermally activated deformation modes under extreme loading.

The transition from Type I to Type III craters with increasing velocity reflects a shift in the dominant energy-absorption mechanism from bulk plastic work to localized thermal dissipation and perforation. This behavior is distinct from that of woven UHMWPE composites or laminated plates, where delamination and fiber breakage often dominate [9,14]. The absence of perforation in Fe-particle impacts, even at velocities up to ~7.4 km/s, highlights the particle-size dependency of penetration resistance, suggesting that UHMWPE films may be particularly effective against sub-micron debris. Moreover, the identification of localized melting in Type II craters suggests that thermal management may be an important consideration in the design of UHMWPE-based protective systems subjected to repeated hypervelocity impacts.

The smooth, reflow-like morphology and localized glossiness observed in the inner zones of Type II craters (e.g., B-6, C-4) are indicative of transient melting followed by rapid solidification, a phenomenon consistent with adiabatic heating under extreme strain rates. While direct compositional evidence (e.g., EDS or Raman spectroscopy) was not acquired in this study, the morphological features align with those reported in prior high-strain-rate polymer impact studies [27,30]. A quantitative energy-balance analysis—partitioning the incident kinetic energy into plastic work, fracture energy, and thermal dissipation—would provide deeper insight into the underlying thermomechanical processes. Such an analysis, however, requires precise single-particle tracking, time-resolved temperature measurements, and complementary computational simulations. Therefore, we intend to pursue these directions in a subsequent integrated study that combines advanced diagnostics with modeling to fully resolve the energy-conversion pathways and material-state changes during hypervelocity impact.

While the present study provides a statistical overview of crater dimensions and a morphology-based classification, a more quantitative scaling analysis—such as plotting crater area or penetration probability against kinetic energy per particle, normalizing by particle diameter, and fitting power-law relations—would offer deeper mechanistic understanding. Such an analysis requires extensive particle-by-particle tracking, precise energy accounting, and potentially complementary simulations to partition energy into plastic work, fracture, and thermal dissipation. We intend to pursue this direction in a subsequent dedicated study, where combined experimental-computational approaches will be employed to establish energy-based scaling laws and further elucidate the constitutive behavior of UHMWPE films under hypervelocity impact.

Additionally, most existing models are validated for strain rates up to ~10^6^ s^−1^, whereas the impacts studied here (≥1 km/s) involve effective strain rates exceeding 10^6^ s^−1^, a regime where thermo-mechanical coupling, adiabatic softening, and ultra-fast chain dynamics become critical. In this extreme regime, conventional constitutive relations may not capture the rapid stress relaxation and thermal-activated flow observed in our experiments. Future work could therefore focus on developing strain-rate- and temperature-sensitive constitutive models specific to UHMWPE films, integrating molecular-dynamics insights with continuum-scale simulations to quantitatively predict the stress states corresponding to the observed Type I → II → III transitions.

## 5. Conclusions

In this study, the damage morphology of UHMWPE films under hypervelocity particle impact was investigated. Mechanical properties of UHMWPE films were obtained via tensile tests, and particle size distributions of Al_2_O_3_ were measured using laser particle analysis. Hypervelocity impact experiments were conducted using a plasma-driven micro-particle accelerator to simulate space debris impacts. Post-impact SEM analysis was performed to examine surface morphology and damage features. By extending the velocity range to ~8.5 km/s and employing micron-scale projectiles, we provide experimental data and mechanistic insights that bridge the gap between macroscopic impact tests and real-world micrometeoroid/orbital-debris threats. The main conclusions are as follows:

(1) Under hypervelocity impact of 40 μm Al_2_O_3_ particles at 0.6–2.7 km/s, the surface of 0.1 mm UHMWPE films exhibited crater/hole diameters ranging from 0 to 44 μm. For 40 μm Al_2_O_3_ particles at 0.6–8.5 km/s, crater/hole diameters ranged from 0 to 48 μm. For 10 μm Al_2_O_3_ particles at 0.6–4.5 km/s, crater/hole diameters ranged from 0 to 30 μm.

(2) Across the three impact velocity conditions, when a sufficient number of Al_2_O_3_ particles were involved, the minimum particle diameter causing penetration can be considered as corresponding to the maximum limiting velocity. For a 0.1 mm UHMWPE film, the penetration particle diameters for Al_2_O_3_ particles at velocities of 2.7 km/s, 4.5 km/s, and 8.5 km/s were 11.35 μm, 4.76 μm, and 2.25 μm, respectively.

(3) Experimental results indicate that UHMWPE films provide effective protection against micrometer-scale hypervelocity Al_2_O_3_ and Fe particles. Based on the morphology of impact craters, three types of microcraters/penetrations can be identified: Type I—low-speed impacts producing craters dominated by plastic deformation; Type II—higher-speed Al_2_O_3_ impacts forming craters with an outer plastic deformation region and an inner region exhibiting plastic fracture and, in some cases, localized melting; Type III—perforation craters formed by very high-speed Al_2_O_3_ particles that fully penetrate the UHMWPE film.

The observed damage modes can be linked to the underlying microstructure of highly oriented UHMWPE films. The aligned molecular chains and reduced entanglement facilitate large-scale plastic drawing and fibrillation, enabling efficient energy dissipation through chain stretching and micro-fibril formation. The pronounced strain hardening evident in tensile tests further contributes to sustained load-bearing capacity under dynamic loading, delaying perforation. At higher impact energies, localized adiabatic heating leads to thermal softening and melting, which absorbs additional kinetic energy but also marks the onset of perforation.

Practical implications for spacecraft shielding design emerge from these insights. For instance, UHMWPE films could serve as an intermediate energy-absorbing layer in hybrid multilayer systems, where a ceramic or metallic bumper layer first fragments incoming projectiles, and the UHMWPE film then captures the debris cloud via plastic deformation and fibrillation. Further performance gains may be achieved by incorporating graphene or other nano-reinforcements to enhance thermal conductivity, reduce localized heating, and improve erosion resistance. While the present study focuses on monolithic film response, these design concepts provide a direct pathway for developing next-generation lightweight, deformable shielding systems for long-duration space missions.

Nevertheless, current research primarily focuses on hypervelocity particle impact experimental methods and damage mechanism analysis, though certain limitations remain. Future directions may include developing a high-strain-rate constitutive model and finite element method for numerical simulations [30,31], conducting molecular dynamics studies to explore microscopic fracture mechanisms [32], designing metamaterial functional structures to enhance protective capabilities [33], and even creating impact testing equipment capable of simulating real space environments [34].

## Figures and Tables

**Table 1:** UHMWPE hypervelocity impact test parameters.

Experiments	Particle Size (μm)	Sample Placement	Speed (km/s)
A0	40	No	0.6–2.7
A	40	Yes	0.6–2.7
B0	40	No	0.6–8.5
B	40	Yes	0.6–8.5
C0	10	No	0.6–4.5
C	10	Yes	0.6–4.5

## Data Availability

The original contributions presented in this study are included in the article. Further inquiries can be directed to the corresponding author.

## References

[1] Rakib M.A., Smith S.T., Tafsirojjaman T. (2024). A review of shielding systems for protecting off-earth structures from micrometeoroid and orbital debris impact. Acta Astronaut..

[2] Pai A., Divakaran R., Anand S., Shenoy S.B. (2022). Advances in the whipple shield design and development: A brief review. J. Dyn. Behav. Mater..

[3] Fowler K., Teixeira-Dias F. (2022). Hybrid shielding for hypervelocity impact of orbital debris on unmanned spacecraft. Appl. Sci..

[4] Li H. (2010). Study on the Impact Effects of Microscopic Space Debris. Ph.D. Thesis.

[5] Wang Y. (2024). Particle-target interactions of high-speed microparticle impact for resulting material modifications. Mater. Today Commun..

[6] Veysset D., Lee J.H., Hassani M., Kooi S.E., Thomas E.L., Nelson K.A. (2021). High-velocity micro-projectile impact testing. Appl. Phys. Rev..

[7] Rogers J.A., Mead P.T., Harrison K., Lukasik G.D., Kota K.R., Kulatilaka W.D., Wilkerson J.W., Lacy T.E. (2022). Hypervelocity impact response of monolithic UHMWPE and HDPE plates. Int. J. Impact Eng..

[8] Rogers J., Mead P.T., Harrison K., Kota K.R., Leaverton J.D., Lukasik G., Kulatilaka W.D., Wilkerson J.W., Lacy T.E. Hypervelocity impact response of polyethylene plates. Proceedings of the AIAA Scitech 2021 Forum.

[9] Xu H., Yu D., Cui J., Shi Z., Song D., Miao C. (2023). The Hypervelocity impact behavior and energy absorption evaluation of fabric. Polymers.

[10] Bian J., Dai K., Lv X., Huang Z., Wu G., Zhang Y. (2024). Effect of Material and Structure of Ultra-High-Molecular-Weight Polyethylene Body Armor on Ballistic Limit Velocity: Numerical Simulation. Polymers.

[11] Kim T., Drakopoulos S.X., Ronca S., Minnich A.J. (2022). Origin of high thermal conductivity in disentangled ultra-high molecular weight polyethylene films: Ballistic phonons within enlarged crystals. Nat. Commun..

[12] Zhang Z., Kang X., Jiang Y., Cai Z., Li S., Cui D. (2023). Access to disentangled ultrahigh molecular weight polyethylene via a binuclear synergic effect. Angew. Chem. Int. Ed..

[13] Liu X., Zhang W., Zhang X., Zhou Z., Wang C., Pan Y., Hu B., Liu C., Pan C., Shen C. (2024). Transparent ultrahigh-molecular-weight polyethylene/MXene films with efficient UV-absorption for thermal management. Nat. Commun..

[14] Mansoori H., Zakeri M., Guagliano M. (2022). Energy absorption and damage mechanism of UHMWPE-aluminum composite sandwich laminate under impact loading: An experimental investigation. J. Sandw. Struct. Mater..

[15] Pan X., Shen L., Schenning A.P.H.J., Bastiaansen C.W.M. (2019). Transparent, High-Thermal-Conductivity Ultradrawn Polyethylene/Graphene Nanocomposite Films. Adv. Mater..

[16] Shi J., Yu D., Xu H., Liu L., Miao C. (2023). Strain Rate Effect of UHMWPE and Its Influence on Hypervelocity Impact Performance. Chin. J. High Press. Phys..

[17] Veysset D., Sun Y., Kooi S.E., Lem J., Nelson K.A. (2020). Laser-driven high-velocity microparticle launcher in atmosphere and under vacuum. Int. J. Impact Eng..

[18] Horanyi M., James D., Kempf S., Munsat T., Sternovsky Z. The SSERVI-IMPACT dust accelerator facility at the University of Colorado. Proceedings of the 47th Annual Lunar and Planetary Science Conference.

[19] Shu A., Collette A., Drake K., Grün E., Horányi M., Kempf S., Mocker A., Munsat T., Northway P., Srama R. (2012). 3 MV hypervelocity dust accelerator at the Colorado Center for Lunar Dust and Atmospheric Studies. Rev. Sci. Instrum..

[20] Liu L., Flanagan K., Robinson P., Desai A., Martinez R. (2025). Towards a hypervelocity optical track microparticle accelerator: Theory and initial validation experiments. J. Appl. Phys..

[21] Sun Y. (2021). High-Velocity Microparticle Impact for Analytical Modelling of High-Strain-Rate Mechanics and Material Behavior. Ph.D. Thesis.

[22] Matsubayashi Y., Ito T., Shinoda K., Terashima K., Akedo J. (2022). Optical emission generated by particle impact during aerosol deposition of alumina films. J. Asian Ceram. Soc..

[23] Tiamiyu A.A., Sun Y., Nelson K.A., Schuh C.A. (2021). Site-specific study of jetting, bonding, and local deformation during high-velocity metallic microparticle impact. Acta Mater..

[24] Rahmati S., Veiga R.G., Mostaghimi J., Coyle T., Dolatabadi A. (2024). High speed impact and solid-state deposition of alumina particles: A molecular dynamics study. J. Eur. Ceram. Soc..

[25] Harilal S.S., Kautz E.J., Phillips M.C. (2022). Spatiotemporal evolution of emission and absorption signatures in a laser-produced plasma. J. Appl. Phys..

[26] Zhang H. (2016). Study on the Melt Extrusion Process, Structure, and Properties of UHMWPE Based on Tensile Rheology. Ph.D. Thesis.

[27] Xie M. (2006). Research on the Improvement of Processing Performance and the Structure and Properties of Ultra-High Molecular Weight Polyethylene. Ph.D. Thesis.

[28] Li Y. (2009). Study on the Damage Behavior of Optical Glass by High-Speed Impact of Dust Particles. Master’s Thesis.

[29] Holmquist T.J., Templeton D.W., Bishnoi K.D. (2001). Constitutive modeling of aluminum nitride for large strain, high-strain rate, and high-pressure applications. Int. J. Impact Eng..

[30] Zhao Z., Meng W., Yan B. (2025). Strain Rate-Dependent Nonlinear Fractional Order Modeling of Elastomer Polymers: A Comprehensive Power-Law Approach. Eng. Sci..

[31] Zhang K., Li W., Zheng Y., Yao W., Zhao C. (2020). Dynamic Constitutive Model of Ultra-High Molecular Weight Polyethylene (UHMWPE): Considering the Temperature and Strain Rate Effects. Polymers.

[32] Zhang M., Yu Y., Li L., Zhou H., Gong L., Zhou H. (2024). A molecular dynamics assisted insight on damping enhancement in carbon fiber reinforced polymer composites with oriented multilayer graphene oxide coatings. Microstructures.

[33] Zhao C., Meng Z., Yi J., Chen C.Q. (2025). Auxetic metamaterials with double re-entrant configuration. Int. J. Mech. Sci..

[34] Robles-De-La-Torre G. (2006). The Importance of the sense of touch in virtual and real environments. IEEE Multimed..

